# OsMGD1-Mediated Membrane Lipid Remodeling Improves Salt Tolerance in Rice

**DOI:** 10.3390/plants13111474

**Published:** 2024-05-27

**Authors:** Shasha Li, Lei Hui, Jingchong Li, Yuan Xi, Jili Xu, Linglong Wang, Lina Yin

**Affiliations:** 1 State Key Laboratory of Soil Erosion and Dryland Farming on the Loess Plateau, College of Natural Resources and Environment, Northwest A&F University, Yangling, Xianyang 712100, China; 2019060322@nwafu.edu.cn (S.L.); huilei@nwafu.edu.cn (L.H.); xy1204930009@163.com (Y.X.); 2021060380@nwafu.edu.cn (J.X.); 2 Institute of Soil and Water Conservation, College of Soil and Water Conservation Science and Engineering, Northwest A&F University, Yangling, Xianyang 712100, China; 3 Institute of Soil and Water Conservation, Chinese Academy of Sciences and Ministry of Water Resources, Yangling, Xianyang 712100, China; ljc_123mail@163.com; 4 College of Agronomy, Northwest A&F University, Yangling, Xianyang 712100, China; 18893929221@163.com

**Keywords:** rice, membrane lipid, *OsMGD1*, photosynthesis, salt tolerance

## Abstract

Salt stress severely reduces photosynthetic efficiency, resulting in adverse effects on crop growth and yield production. Two key thylakoid membrane lipid components, monogalactosyldiacylglycerol (MGDG) and digalactosyldiacylglycerol (DGDG), were perturbed under salt stress. MGDG synthase 1 (MGD1) is one of the key enzymes for the synthesis of these galactolipids. To investigate the function of *OsMGD1* in response to salt stress, the *OsMGD1* overexpression (OE) and RNA interference (Ri) rice lines, and a wild type (WT), were used. Compared with WT, the OE lines showed higher chlorophyll content and biomass under salt stress. Besides this, the OE plants showed improved photosynthetic performance, including light absorption, energy transfer, and carbon fixation. Notably, the net photosynthetic rate and effective quantum yield of photosystem II in the OE lines increased by 27.5% and 25.8%, respectively, compared to the WT. Further analysis showed that the overexpression of *OsMGD1* alleviated the negative effects of salt stress on photosynthetic membranes and oxidative defense by adjusting membrane lipid composition and fatty acid levels. In summary, OsMGD1-mediated membrane lipid remodeling enhanced salt tolerance in rice by maintaining membrane stability and optimizing photosynthetic efficiency.

## 1. Introduction

Salt stress is one of the most widespread abiotic stresses limiting crop growth and yield, and the area of salt-affected cropland is increasing year by year due to drastic climate change and farming practices [1,2]. It is estimated that approximately 20% of the cultivated land and 50% of the irrigated farmland worldwide will be affected by salt stress by the middle of this century [3]. In salt-affected farmland, crops take up excess Na^+^ and Cl^−^ ions, causing ion toxicity, osmotic stress, oxidative stress, and severe impairment of photosynthetic capacity, which ultimately lead to a reduction in yield [4,5,6]. However, with the world population projected to reach 9.6 billion by 2050, the challenge of avoiding food shortages requires at least a 70% increase in food production from current levels [7]. Therefore, it is crucial to investigate the mechanisms of salt tolerance in crops to ensure the sustainability of global agriculture.

Rice (*Oryza sativa* L.) is a staple food for nearly half of the world’s population and has been recognized as one of the three major food crops of the world, as well as a critical monocotyledonous model crop [8,9]. Unfortunately, rice is a salt-sensitive crop, and salt stress causes irreversible damage to photosynthetic organs, which undoubtedly significantly inhibits its growth, development, and yield. Hence, there is an urgent need to find a feasible method to reduce the damaging effects of salt stress on rice photosynthesis, and thus increase rice yield. The use of effective transgenic technologies could potentially improve the photosynthetic efficiency of salt-stressed plants. For example, the overexpression of the salt-tolerant chloroplast FBPase gene *PcCFR* in rice has significantly improved salt tolerance by enhancing photosynthetic efficiency [10]. Similarly, wheat *TaLHC86*-silenced plants showed severe impairment in photosynthetic rate and electron transport, resulting in reduced salt tolerance in wheat [11]. Moreover, the overexpression of *ABCB28* and *ABCB29* in *Arabidopsis* has been demonstrated to enhance salt tolerance by increasing photosynthesis [12]. The results suggest that using transgenic technology to improve photosynthesis under salt stress may be an effective strategy for developing new crops with strong salt tolerance in rice.

Photosynthesis and biomass production in plants are based on photosynthetic membranes that are organized into thylakoids inside the chloroplasts [13]. The major lipid components of thylakoid membranes are monogalactosyldiacylglycerol (MGDG), digalactosyldiacylglycerol (DGDG), sulphoquinovosyl diacylglycerol (SQDG), and phospholipid phosphatidylglycerol (PG) [14,15]. Among these, galactolipids (MGDG and DGDG) make up approximately 80% of the total polar lipid content in plastids [15]. They play a crucial role in determining the thylakoid architecture and optimizing photosynthetic function [13]. Crystallographic analysis confirmed MGDG exists within photosystems I and II (PSI, PSII), as well as in the cytochrome *b*6*f* complex (Cyt *b*6*f*), and DGDG is present in PSII [16,17]. In addition, MGDG has a conical shape and therefore has non-bilayer-forming properties, whereas DGDG has a cylindrical shape and is a bilayer-forming lipid [18]. These unique properties make MGDG and DGDG essential for the organization of thylakoid membranes.

MGDG is synthesized by MGDG synthase (MGD), which catalyzes the transfer of galactose from UDP-galactose to diacylglycerol (DAG) [19]. MGDG is also a precursor for DGDG synthesis [20]. Therefore, MGD is essential for MGDG and DGDG synthesis. The plants’ MGD enzymes are classified into type A (MGD1) and type B (MGD2 and MGD3) enzymes based on sequence homology and physiological properties [15,21,22]. *MGD1* is highly expressed in green tissues, whereas *MGD2* and *MGD3* are mainly expressed in non-photosynthetic tissues such as roots and flowers [21,23]. Functional studies have shown that *MGD1* is important for thylakoid membrane stabilization and chloroplast biogenesis. For example, a previous study showed that the absence of MGDG and DGDG in *mgd1* and *amiR-MGD1* mutants in *Arabidopsis* resulted in the complete impairment of photosynthetic membranes and photosynthetic pigment transforming capacity, and therefore inhibited plant growth [24,25,26,27]. Similarly, *opaque5*, a maize mutant with a loss-of-function in MGD1, exhibits a flawed membrane structure and pale green seedlings [15]. Moreover, *Arabidopsis mgd1* mutants exhibited heightened sensitivity to light and aluminum stress [24,28]. In rice, the function of *OsMGD2* (type B) in regulating rice anther development and seed quality is well-established [22]. Furthermore, *OsMGD2* overexpression lines improved salt tolerance in rice by maintaining Na^+^ and K^+^ balance in shoots and roots [29]. Also, the overexpression of *OsMGD2* from submergence-tolerant rice (“FR13A”) showed improvement in salt tolerance [30]. Previous studies have shown significant upregulations of *Arabidopsis MGD1* and *Dendrobium catenatum MGD1* in response to salt stress [31,32]. However, it remains unclear whether *OsMGD1* also plays a role in enhancing salt tolerance in rice.

Despite having similar sequences, *OsMGD1* (leaves) and *OsMGD2* (anthers and seeds) exhibit distinct expression patterns in various rice tissues [22], indicating their roles may be different under salt stress. Also, there are still limited data on increasing plant salt stress defense through the protection and improvement of photosynthesis [4]. Therefore, these factors prompted us to explore the function of *OsMGD1* under salt stress. Here, we have shown that *OsMGD1* could positively regulate salt tolerance in rice. A higher expression of *OsMGD1* regulated membrane lipid remodeling, which reduced the adverse effects of salt stress on photosynthetic membranes and capacity. Our study provides valuable information for breeding salt-tolerant crops.

## 2. Results

### 2.1. OsMGD1 Enhanced Growth of Salt-Stressed Rice Seedlings

**Figure 1:**
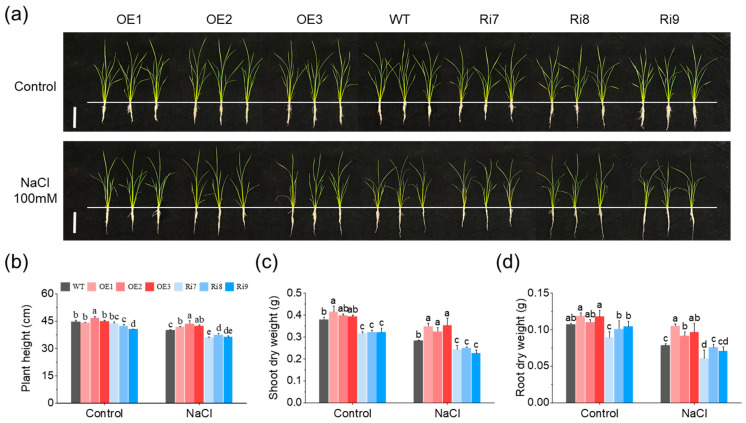
Effect of *OsMGD1* transformation on rice seedling growth under salt stress. (**a**) Phenotypic comparison of wild-type and *OsMGD1* transgenic plants grown with either 0 mM NaCl (Control) or 150 mM NaCl. Images were taken 6 d after salt treatment. Bar = 10 cm. (**b**–**d**) Plant height (**b**), shoot dry weight (**c**), and shoot dry weight (**d**) of wild-type and *OsMGD1* transgenic rice plants under normal growth and salt stress. WT, wild type; OE, *OsMGD1-*overexpressing lines; Ri, *OsMGD1*-RNA interfering lines. Data are means ± SD (*n* = 5 biological replicates). Different letters indicate significance at *p* < 0.05 as determined by LSD test.

To verify the function of the *OsMGD1* gene under salt stress conditions, the growth of rice seedlings was observed after 6 days of NaCl treatment. Under a normal condition (0 mM NaCl), there was no significant difference in the growth of overexpressed *OsMGD1* rice lines (OE1, OE2 and OE3) compared with the WT, but the aboveground growth of RNA interference lines (Ri7, Ri8 and Ri9) was inhibited (Figure 1a). The fresh weight (FW) and dry weight (DW) of the shoot were significantly lower in the Ri lines compared to the WT, validating the phenotypic results (Figure 1b,c and Figure S1a). In contrast, rice seedlings of OE lines exposed to salt treatment (100 mM NaCl) showed better growth and greener leaves compared to the WT, whereas Ri seedlings exhibited evident symptoms of salt stress (withered leaves and dwarfed plants) (Figure 1a). The plant height and shoot weight were also significantly higher in the OE lines and lower in the Ri lines compared to the WT under the salt stress condition (Figure 1b,c and Figure S1a). Specifically, the plant height increased by 6.4% in the OE lines, while it decreased by 8.4% in the Ri plants (Figure 1b). Similarly, the shoot dry weight increased by 21.1% in OE lines, while it decreased by 15.3% in Ri lines (Figure 1c).

Furthermore, the root fresh and dry weights of *OsMGD1* transgenic lines did not differ from those of the WT values under the normal condition (Figure 1d and Figure S1b). However, under salt stress, the biomass of the root was prominently changed in the OE plants, with 19.2% and 24.4% increases in FW and DW compared to WT, respectively (Figure 1d and Figure S1b). In addition, the total fresh and dry weights of the Ri lines of *OsMGD1* were significantly lower than those in the WT under either normal or salt stress conditions (Supplementary Figure S1c,d). Although the total fresh and dry weights of the OE lines did not change significantly under normal conditions, they exhibited a significant increase compared to the WT under the salt stress condition (Supplementary Figure S1c,d).

### 2.2. Overexpression of OsMGD1 Increased Photosynthetic Capacity under Salt Stress

**Figure 2:**
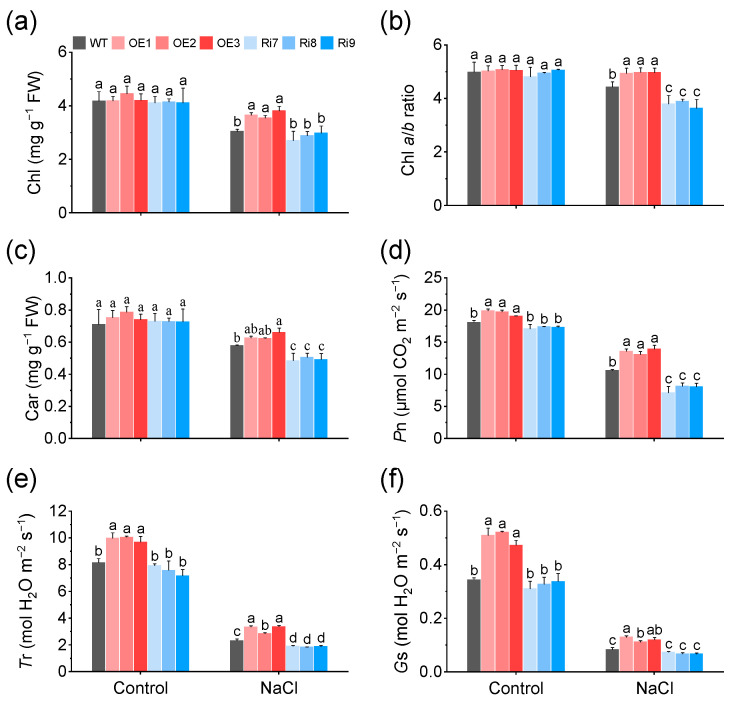
Changes in pigment content and photosynthetic parameters of rice *OsMGD1* transgenic lines under salt stress. (**a**–**c**) Photosynthetic leaf pigments analysis: (**a**) total chlorophyll (Chl), (**b**) chlorophyll a/b ratio (Chl *a*/*b*), and (**c**) carotenoid content (Car). Data are means ± SD (*n* = 4 biological replicates); (**d**–**f**) Photosynthetic parameters include (**d**) net photosynthetic rate (*P*n), (**e**) transpiration rate (*T*r), and (**f**) stomatal conductance (*G*s). WT, wild type; OE, *OsMGD1-*overexpressing lines; Ri, *OsMGD1*-RNA interfering lines. Data are means ± SD (*n* = 5 biological replicates). Different letters indicate significance at *p* < 0.05 as determined by LSD-test.

Leaves of rice lines overexpressing *OsMGD1* were much greener than WT leaves under salt stress. Therefore, their chlorophyll (Chl) content was estimated. As shown in Figure 2a–c, NaCl treatment significantly decreased the photosynthetic pigment content of rice seedlings, but the Chl content of the OE lines increased by 20.8% compared to WT, respectively. In addition, under salt stress, the Chl *a*/*b* ratio increased significantly in OE lines and decreased significantly in Ri lines compared to WT (Figure 2b). The above results are consistent with the phenotype of these lines. Further analysis revealed that the increase in Chl content and Chl *a*/*b* ratio in OE lines under salt stress were mainly due to a significant increase in Chl *a* content (Supplementary Figure S2a,b). Additionally, under salt stress, the carotenoid (Car) content was significantly increased in the OE lines but significantly decreased in the Ri lines compared to the WT (Figure 2c).

Increased photosynthetic pigment content is thought to potentially confer higher photosynthetic capacity to plants. Thus, photosynthetic parameters of leaves of WT and *OsMGD1* transgenic rice lines were determined. The results showe that the OE plants had a significantly increased net photosynthetic rate (*P*n), and transpiration rate (*T*r) in response to salt stress compared with the WT, and the Ri lines had the opposite effect (Figure 2d,e). Among them, the *P*n was 27.5% higher in OE plants compared to WT, and 26.6% lower in Ri lines compared to WT. Also, the stomatal conductance (*G*s) of the OE lines was significantly higher than that of the WT (Figure 2f). In addition, *OsMGD1* had no significant effect on the intercellular CO_2_ concentration (*C*i) under normal conditions and salt stress (Supplementary Figure S2c).

### 2.3. Effects of OsMGD1 on Chlorophyll Fluorescence under Salt Stress

**Figure 3:**
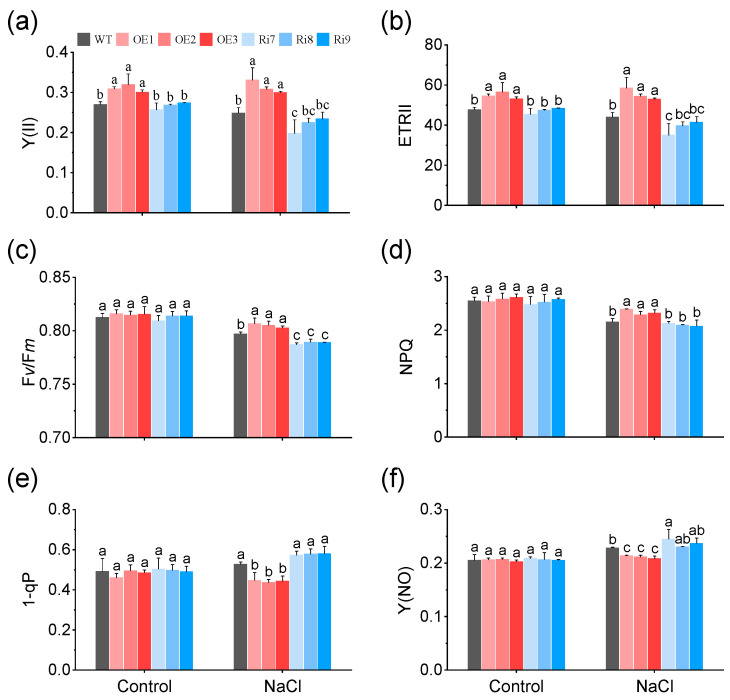
Chlorophyll fluorescence parameters of *OsMGD1* under salt stress. Leaves were dark-adapted for 30 min before measuring the induction of fluorescence. Chlorophyll fluorescence of photosystem II (PSII) was measured, including (**a**) effective quantum yield of PSII, Y(II); (**b**) electron transport rate through PSII, ETRII; (**c**) maximum quantum yield of PSII, F*v*/F*m*; (**d**) non-photochemical quenching, NPQ; (**e**) the redox state of the QA electron acceptor of PSII, 1-qP; (**f**) quantum yield of non-regulated non-photochemical energy loss in PSII, Y(NO). WT, wild type; OE, *OsMGD1-*overexpressing lines; Ri, *OsMGD1*-RNA interfering lines. Data are means ± SD (*n* = 5 biological replicates). Different letters indicate significance at *p* < 0.05 as determined by LSD test.

To understand how *OsMGD1* affected the biomass of rice seedlings under salt stress, photosynthetic efficiency was further assessed using chlorophyll fluorescence measurements. Under normal growth condition, the plants of OE showed a significant increase in the effective photosystem II (PSII) quantum yield [Y(II)] and the PSII electron transport rate (ETRII) compared to WT (Figure 3a,b). In addition, salt stress significantly inhibited the PSII photochemical efficiency of WT and Ri rice seedlings, resulting in a decrease in the Y(II), ETRII, maximum quantum yield of PSII (F*v*/F*m*), and non-photochemical quenching (NPQ) (Figure 3a–d). Conversely, overexpressing *OsMGD1* plants improved resilience to salt stress, maintained superior fluorescence parameters, and significantly increased values in Y(II), ETRII, F*v*/F*m*, and NPQ compared to the WT (Figure 3a–d). Among them, the Y(II) was notably increased in the OE plants by 25.8%, and decreased in the Ri lines by 11.9%, compared to WT (Figure 3a). Moreover, under salt treatment, the OE plants showed the QA electron acceptor of PSII (1-qP) and non-regulated non-photochemical energy loss in PSII [Y(NO)] decreased by 16.1% and 7.5% in OE plants compared with WT (Figure 3e,f).

### 2.4. Regulation of Na^+^/K^+^ Balance by Overexpression of OsMGD1 Conferred Salt Tolerance

**Figure 4:**
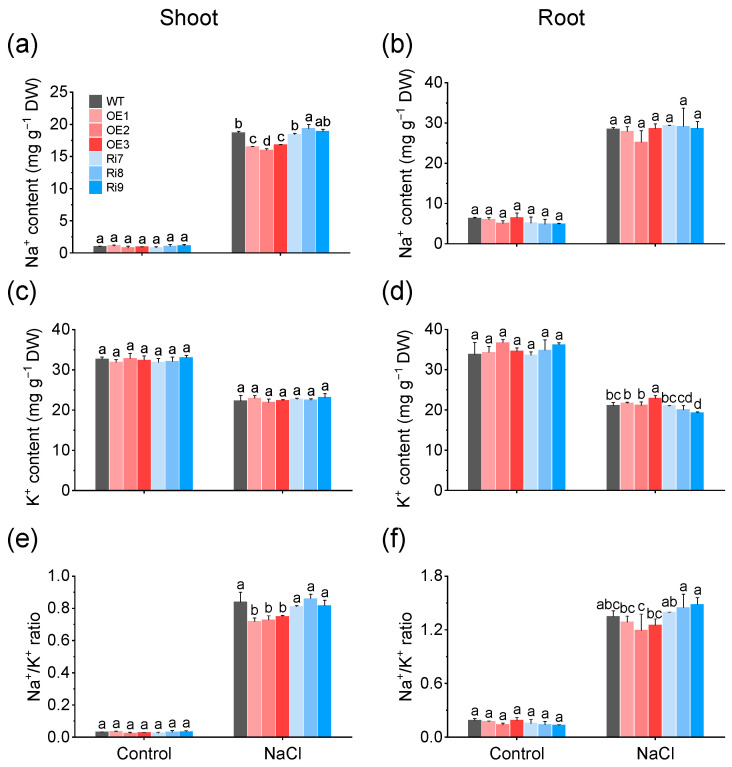
Regulation of Na^+^ and K^+^ content and Na^+^/K^+^ ratio in rice shoot (**left**) and root (**right**) under salt stress by *OsMGD1*. (**a**,**b**) Na^+^ content; (**c**,**d**) K^+^ content, and (**e**,**f**) Na^+^/K^+^ ratio in rice seedlings of WT and *OsMGD1* transgenic lines under non-salt condition (Control) or 100 mM NaCl stress (6 d). WT, wild type; OE, *OsMGD1-*overexpressing lines; Ri, *OsMGD1*-RNA interfering lines. Data are means ± SD (*n* = 5 biological replicates). Different letters indicate significance at *p* < 0.05 as determined by LSD test.

Salt tolerance in plants is related to the avoidance of Na^+^ accumulation and the maintenance of the Na^+^/K^+^ ratio. Hence, Na^+^ and K^+^ contents were assessed in WT and *OsMGD1* transgenic rice lines under normal (0 mM NaCl) and salt stress (100 mM NaCl, 6 days) conditions. Under the normal condition, the contents of Na^+^, K^+^, and Na^+^/K^+^ ratios were similar in shoots and roots across all lines (Figure 4). Compared to WT, the overexpressed lines OE1, OE2, and OE3 showed a significant decrease in Na^+^ content in shoots under salt stress, with reductions of 12.1%, respectively (Figure 4a). In roots, the Na^+^ content of *OsMGD1* transgenic lines was not different from WT (Figure 4b). Additionally, salt stress significantly reduced the K^+^ content in the shoots and roots of all lines compared to normal condition (Figure 4c,d). Under salt stress, the OE3 line exhibited a significant increase in root K^+^ content compared to the WT. But among three Ri transgenic lines (Ri7, Ri8, and Ri9), only the Ri9 lines showed a notable decrease (Figure 4b). Meanwhile, salt treatment also resulted in increased Na^+^/K^+^ ratios in both shoots and roots of rice seedlings compared to normal condition (Figure 4e,f). Under salt stress, the Na^+^/K^+^ ratios of shoots of OE lines were significantly reduced by 12.7%, respectively, compared with WT (Figure 4e). Similarly, no significant differences in root Na^+^/K^+^ ratios were observed between transgenic and WT plants (Figure 4f).

### 2.5. OsMGD1-Mediated Membrane Lipid Remodeling Positively Regulates Rice Salt Tolerance

**Figure 5:**
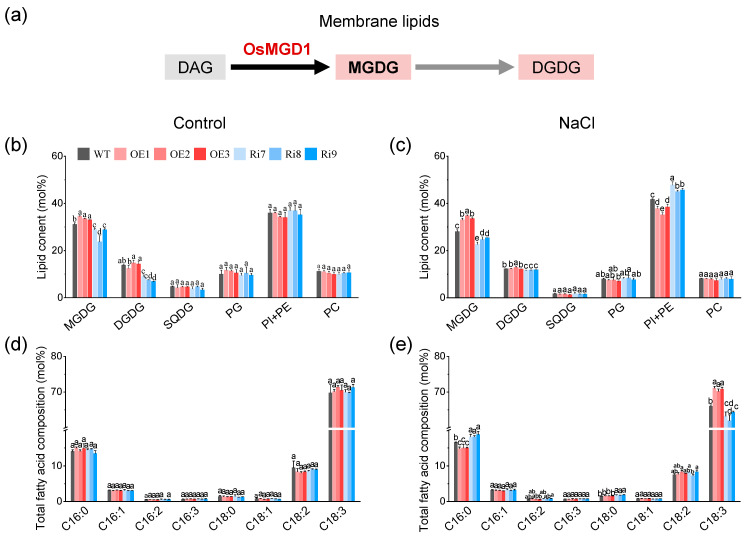
Membrane lipid content and total fatty acid composition of WT and *OsMGD1* transgenic rice seedlings under salt stress. (**a**) OsMGD1 mediates the biosynthetic pathway of the galactolipid MGDG and DGDG in rice. OsMGD1 uses DAG as a substrate to synthesize MGDG, and MGDG is a precursor of DGDG. MGDG and DGDG are the hallmark lipids of photosynthetic membranes, accounting for approximately 80% of total membrane lipids. (**b**,**c**) Relative amounts of lipids and (**d**,**e**) fatty acid composition of total lipids in leaves of rice seedlings under normal (control, **left**) and salt stress (NaCl, **right**) condition. MGDG, monogalactosyldiacylglycerol; DGDG, digalactosyldiacylglycerol; SQDG, sulfoquinovosyldiacylglycerol; PG, phosphatidylglycerol; PI, phosphatidylinositol; PE, phosphatidylethanolamine; PC, phosphatidylcholine. WT, wild type; OE, *OsMGD1-*overexpressing lines; Ri, *OsMGD1*-RNA interfering lines. Data are means ± SD (*n* = 5 biological replicates). Different letters indicate significance at *p* < 0.05 as determined by LSD test.

In rice, *OsMGD1* synthesizes MGDG using DAG as a substrate, and MGDG is a precursor for DGDG synthesis (Figure 5a). To further investigate the mechanism by which *OsMGD1* improves salt tolerance in transgenic rice seedlings, we analyzed the lipid content and fatty acid composition of leaves from WT and *OsMGD1* transgenic lines (Figure 5b–e). Under the normal condition, *OsMGD1* significantly affected the biosynthesis of MGDG and DGDG. Among them, MGDG content in OE lines was significantly increased by 8.5%, and in Ri plants it was significantly decreased by 12.6%, compared with WT (Figure 5b). Similarly, the DGDG content was also dramatically reduced in the Ri lines (42.8%) compared to the WT (Figure 5b). Under salt stress, the MGDG content was 20.3% higher in OE lines compared to WT (Figure 5c). Also, MGDG and DGDG contents were significantly lower in the Ri plants, at 13.7% and 4.2%, respectively (Figure 5c). In contrast, phospholipid (PI and PE) content decreased by 10.9% in the OE lines and increased by 10.7% in the Ri plants relative to WT (Figure 5c). In addition, no obvious differences in SQDG, PG, and PC contents were observed between the WT and transgenic lines under salt stress.

**Figure 6:**
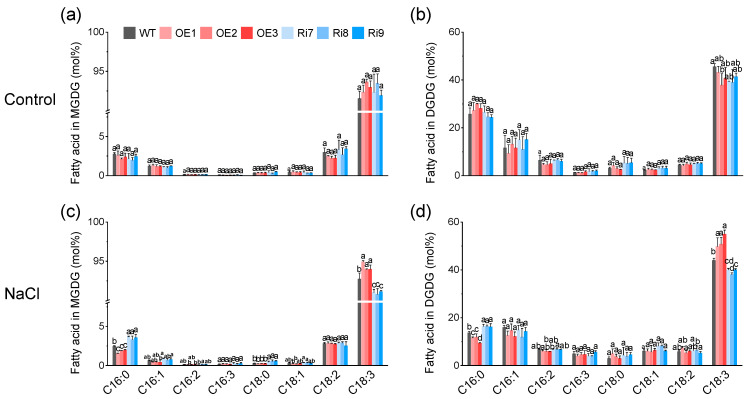
Changes in the fatty acid composition of MGDG and DGDG in leaves of WT and *OsMGD1* transgenic rice seedlings under (**a**,**b**) control (0 mM NaCl) and (**c**,**d**) salt treatment (100 mM NaCl) conditions. MGDG, monogalactosyldiacylglycerol; DGDG, digalactosyldiacylglycerol. WT, wild type; OE, *OsMGD1-*overexpressing lines; Ri, *OsMGD1*-RNA interfering lines. Data are means ± SD (*n* = 5 biological replicates). Different letters indicate significance at *p* < 0.05 as determined by LSD-test.

Next, we also compared the fatty acid composition of total lipids and galactolipids (MGDG and DGDG) between WT and *OsMGD1* rice seedlings. In rice leaves, C18:3 was the main fatty acid component (Figure 5d,e and Figure 6). Under normal condition, there were no significant changes in fatty acid composition between WT and transgenic plants (Figure 5d and Figure 6a,b). Under salt stress, the C18:3 content in the total lipids of OE lines significantly increased by 7.1% compared to WT, while in Ri plants it decreased by 6.6% (Figure 5e). Conversely, under salt stress, the palmitic acid (C16:0) content in total lipids of OE lines significantly decreased by 9.8% compared to WT, while in Ri plants it significantly increased by 10.8% (Figure 5e). Similarly, in OE plants, galactolipids (MGDG and DGDG) showed a significant increase in C18:3 and a significant decrease in C16:0, while Ri plants showed the opposite results (Figure 6c,d). Additionally, the Ri lines exhibited a significant increase in stearic acid (C18:0) content in both total lipids and MGDG under salt stress when compared to the WT (Figure 5e and Figure 6c).

### 2.6. OsMGD1 Increased Membrane Fluidity and Reduced Membrane Damage

**Figure 7:**
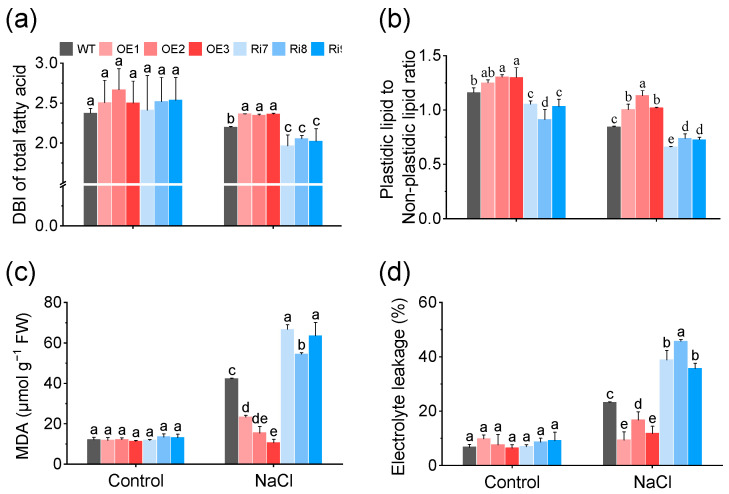
Effect of MGD1 on DBI (**a**), plastidic lipids (MGDG, DGDG, and PG) to non-plastidic lipids (phospholipids other than PG) ratio (**b**), MDA content (**c**), and electrolyte leakage (**d**) in rice seeding exposed to salt stress. DBI, double-bond index; MDA, malondialdehyde. WT, wild type; OE, *OsMGD1-*overexpressing lines; Ri, *OsMGD1*-RNA interfering lines. Data are means ± SD (*n* = 5 biological replicates). Different letters indicate significance at *p* < 0.05 as determined by LSD test.

The double bond index (DBI) parameter characterizes the average extent of fatty acid unsaturation and reflects membrane fluidity. No significant difference in DBI was observed between WT and *OsMGD1* transgenic rice seedlings under normal condition, which is consistent with the changes in fatty acid content (Figure 7a and Figure S3). Under salt stress, OE lines exhibited higher membrane fluidity (higher DBI) compared with WT, while Ri lines showed lower membrane fluidity (lower DBI) (Figure 7a and Figure S3). Specifically, the DBI of total lipids, MGDG, and DGDG increased by 7.3%, 1.9%, and 9.7% in the OE lines compared with WT, and decreased by 8.3%, 1.5%, and 7.4% in the Ri plants, respectively (Figure 7a and Figure S3).

On the other hand, salt stress caused damage to the photosynthetic membrane, as demonstrated by a significant decrease in the ratio of plastidic lipids (MGDG, DGDG, and PG) to non-plastidic lipids (phospholipids other than PG). Our results show that this ratio was reduced by 13.8% in Ri plants compared with WT under the normal condition (Figure 7b), while this ratio increased by 18.8%, 34.1%, and 20.7% in OE plants and decreased by 21.8%, 12.3%, and 13.8% in Ri lines compared with WT under salt stress (Figure 7b).

In addition, malondialdehyde (MDA) and electrolyte leakage (EL) contents serve as reliable indicators of membrane damage induced by abiotic stress. Similarly, we found that salt stress caused a notable increase in MDA and EL contents (Figure 7c,d). Compared to WT, OE plants exhibited notably lower MDA (61.0%) and EL contents (45.5%), while these values were significantly higher in the Ri lines, with increases of 72.8% and 45.4%, respectively (Figure 7c,d).

## 3. Discussion

**Figure 8:**
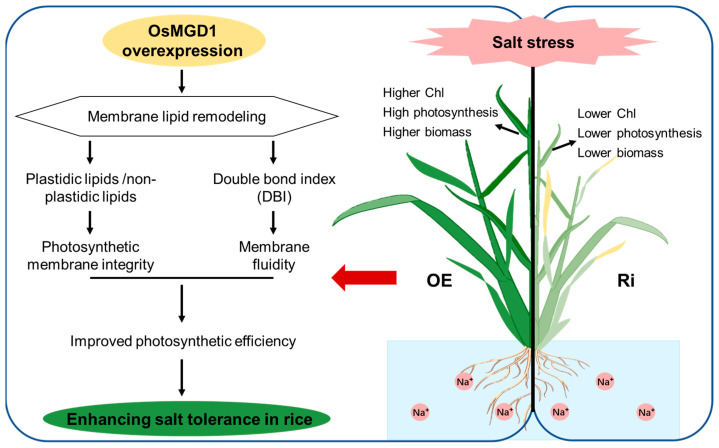
Model diagram of OsMGD1-mediated membrane lipid remodeling to enhance salt tolerance in rice. Overexpression of *OsMGD1* increases the ratio of plastidic to non-plastidic lipids and the double bond index (DBI) in rice leaves. This compensates for chloroplast lipid deficiencies and preserves the structure and fluidity of photosynthetic membranes under salt stress. Meanwhile, the lipid remodeling driven by *OsMGD1* improved chlorophyll levels and photosynthetic performance under stress, mitigating its detrimental impact on rice seedling growth.

Soil salinization threatens rice production and future food security. Consequently, there is an urgent need to understand the mechanisms by which crops respond to salt stress and to develop salt-tolerant rice with high yield potential, addressing the food demands of the growing global population. In recent years, advancements in gene editing technology have significantly increased the potential for enhancing crop salt tolerance. Lipid remodeling is pivotal for plant salt tolerance as it directly impacts membrane integrity, fluidity, and the function of membrane proteins [33]. Moreover, plant salt tolerance is well-established to be intricately associated with the regulation of photosynthesis, yet the potential for plant photosynthetic activity under salt stress is largely unexplored. Previous studies revealed that *OsMGD2* positively regulates salt tolerance in rice mainly by maintaining Na^+^/K^+^ balance in shoots and roots [29]. In this study, we investigated the role of type A *OsMGD1*, a gene that is highly expressed in the photosynthetic tissues (leaves) of rice. We found that *OsMGD1* mediates membrane lipid remodeling to enhance photosynthesis and salt tolerance in rice seedlings under salt stress. As shown in Figure 8, the ratio of plastidic to non-plastidic lipids in leaves of *OsMGD1-*overexpressing plants increased under salt stress to replenish chloroplast lipids and maintain the integrity of photosynthetic membranes. Meanwhile, the increase in DBI in OE plants indicated enhanced membrane fluidity in response to salt stress. Ultimately, *OsMGD1* increased chlorophyll content and photosynthetic efficiency, mitigating the impact of salt stress on biomass. These results offer preliminary insights into the regulatory mechanism of *OsMGD1* in response to salt stress and further support the effectiveness of membrane lipid remodeling in enhancing salt tolerance in rice.

### 3.1. OsMGD1 Enhanced Salt Tolerance in Rice by Remodeling Membrane Lipids

In plants, MGDG and DGDG are highly conserved and are major components of plastidic and chloroplast membranes, playing crucial roles in preserving membrane integrity and stability [15]. MGDG has an intrinsic tendency to form inverse hexagonal lipid phases, which maintains membrane stability by creating high internal lateral pressure among the fatty acyl chains and exerting pressure on the membrane proteins [34]. Higher MGDG and DGDG levels also help to reduce the loss of chloroplast membrane lipids, safeguarding chloroplast integrity and mitigating damage to photosynthesis [20,35]. Previous studies have indicated that these lipids play a crucial role in the photosynthetic efficiency and membrane integrity of *Arabidopsis* [20,27]. Furthermore, Wang et al. [30] and Ge et al. [36] found that salt stress reduces galactose levels in photosynthetic membranes, impairing photosynthesis and consequently hindering plant growth. Our study results amply confirm that increasing MGDG and DGDG content contributes to improved plant salt tolerance. In this study, we found that the overexpression of *OsMGD1* increased MGDG content in rice seedlings and led to better growth under salt stress. Conversely, plants with suppressed *OsMGD1* expression suffered from a lack of both MGDG and DGDG, leading to noticeable salt stress symptoms like leaf curling and yellowing (Figure 1 and Figure 5). The apparent differences in the above phenotypes might be attributed to the increased MGDG and DGDG contents, which protect the function of photosynthetic membranes. Furthermore, the overexpression of *OsMGD1* increased the content of galactolipids relative to WT, while it significantly reduced the content of phospholipids (PE and PI) (Figure 5). These findings imply that *OsMGD1* might facilitate the synthesis of galactolipids by modulating the glycerolipid pathway between the cytosolic and plastidic compartments under salt stress.

Moreover, the lipid composition of plant membranes, specifically the balance between plastidic lipids (MGDG, DGDG, SQDG, PG) and nonplastidic lipids (PC, PE, PI), reflects the extent of damage to the photosynthetic membranes [14,15,36]. Our study shows that salt stress severely impaired the photosynthetic membranes of rice seedling leaves, as evidenced by a decreased ratio of plastidic to non-plastidic lipids (Figure 7). However, the overexpression of *OsMGD1* resulted in a significant increase in the ratio of plastidic to nonplastidic lipids compared to the WT under salt stress (Figure 7). This effect was also confirmed in the Ri plants (Figure 7). Additionally, the overexpression of *OsMGD1* promoted fatty acid unsaturation under salt stress, mainly attributed to changes in the content of C18:3 (Figure 5 and Figure 6). Higher unsaturation levels led to increased membrane fluidity, which improved salt tolerance. This was observed in the OE lines with a high DBI (Figure 7). In contrast, the Ri plants had lower membrane fluidity due to a low DBI. These results suggest that *OsMGD1* modified membrane lipid and fatty acid composition, which helped mitigate the impact of salt stress on rice seedling photosynthetic membranes.

### 3.2. Photosynthetic Capacity of Rice under Salt Stress Was Maintained by OsMGD1

The photosynthetic pigment is a crucial indicator of photosynthetic capacity [37]. In this study, we found that OE lines maintained higher levels of total Chl content and Chl *a*/*b* ratio under salt stress relative to WT, mainly due to a higher level of Chl *a* (Figure 2 and Figure S2). Chl *a* is crucial for electron transfer and capturing light energy in photosynthesis, while Chl *b* assists in light absorption [37]. The variation of Chl content in *OsMGD1* transgenic lines paralleled the changes observed in *OsMGD2* transgenic plants under salt stress [29], implying salt-induced membrane lipid content alterations mainly affect Chl *a*. In addition, carotenoids participate in multiple activities within plant cells, such as acting as antioxidants against reactive oxygen species (ROS), forming integral parts of the light-harvesting complex, and functioning as hormonal precursor molecules for stress response and development [38]. We found that suppressing Os*MGD1* expression significantly reduces carotenoid levels during salt stress (Figure 2), possibly attributable to a reduced capacity for interfacial curvature resulting from decreased MGDG level, which directly impacted the membrane’s ability to bind carotenoids [39]. More importantly, the unsaturation of chloroplast lipids occurred simultaneously with photoprotection by carotenoid pigments to enhance photosynthetic capacity under salt stress [40].

Salt stress commonly causes reduced water uptake in leaves, which limits stomatal opening and blocks electron transfer; together, these effects reduce photosynthetic efficiency and can even lead to plant death [4,6]. Despite some salt-tolerant plants reducing transpiration through lower stomatal conductance to conserve water, this usually impairs photosynthetic CO_2_ assimilation [41]. Our results show that rice seedlings exhibit notably reduced *P*n, *T*r, and *G*s parameters under salt stress (Figure 2). Nevertheless, the up-regulation of *OsMGD1* expression was able to alleviate the salt stress-induced reduction in CO_2_ assimilation rate, stomatal conductance, and transpiration rate, thus reducing the photosynthetic capacity losses caused by salt stress (Figure 2). Recent studies also support our findings in *Arabidopsis*, where the overexpression of *ABCB28* and *ABCB29* could enhance plant salt tolerance by increasing photosynthesis [12].

In addition, MGDG and DGDG are essential components within the lipid bilayer of the thylakoid membrane, playing a direct role in photosynthesis by distributing in photosynthetic complexes like PSII, PSI, and Cyt *b*6*f*, as well as by providing membrane stability under stress [16,17]. Previous studies have found that salt stress significantly reduced several chlorophyll fluorescence parameters such as Y(II), F*v*/F*m*, ETR, and photochemical quenching (qP) [42]. Our study found that overexpressing *OsMGD1* in OE lines enhanced Y(II), ETRII, and F*v*/F*m*, implying better light energy utilization through improved electron transport and photochemical efficiency (Figure 3). Equally important, the low levels of 1-qP and Y(NO) in OE plants indicated the strong electron transfer activity of PSII (Figure 3). Therefore, we conclude that *OsMGD1* overexpression could mitigate photosystem damage under salt stress by remodeling membrane lipids, which ultimately support biomass growth in rice seedlings.

### 3.3. OsMGD1 Improved Antioxidant Capacity and Maintained Shoot Na^+^/K^+^ Homeostasis under Salt Stress

Salt stress is known to cause lipid peroxidation and cell membrane damage in plants. In rice, enhanced expression of the *ERF1* and *STK* genes has been associated with increased antioxidant capacity and improved salt resistance [43,44]. Our study found that overexpressing the *OsMGD1* gene could mitigate damage from salt-induced lipid peroxidation and cell membrane damage (Figure 7). This protective effect was likely due to *OsMGD1*-mediated membrane lipid remodeling, which enhanced membrane fluidity and contributed to the reductions in lipid peroxidation and electrolyte leakage under salt stress.

Maintaining the Na^+^/K^+^ balance is important for plant adaptation to salt stress, as high Na^+^ levels can cause K^+^ efflux and disrupt cellular osmotic equilibrium [45]. In rice, specific proteins such as *OsCYB5* and *OsRFPHC-13* play key roles in Na^+^/K^+^ homeostasis, enhancing salt tolerance [45,46]. Our study reveals that the overexpression of the *OsMGD1* gene results in a reduced Na^+^/K^+^ ratio, mainly achieved by lowering Na^+^ accumulation in shoots. However, inhibiting gene expression did not notably impact the above parameters (Figure 4). Furthermore, *OsMGD1* appeared to have little impact on root Na^+^ and K^+^ contents (Figure 4). This finding is consistent with the function of Pumpkin *CmHKT1;1,* which reduces shoot Na^+^ to maintain a favorable Na^+^/K^+^ ratio under salt stress conditions [47]. In addition, overexpression lines of *OsMGD2*, which belongs to the same family as *OsMGD1*, showed a more pronounced reduction in Na^+^/K^+^ ratios in both shoots and roots [29]. Compared with *OsMGD2*, *OsMGD1* enhanced rice salt stress tolerance mainly by enhancing leaf antioxidant capacity and maintaining shoots Na^+^/K^+^ homeostasis.

## 4. Materials and Methods

### 4.1. Plant Materials and Salt Stress Treatment

The rice (*Oryza sativa* L.) *Japonica* cultivar *Nipponbare* was used as the wild type (WT). The *OsMGD1* (Accession: Os09g0423600) rice transgenic lines were obtained from previous studies, including three independent overexpression lines (OE1, OE2, and OE3) and RNA interference lines (Ri7, Ri8, and Ri9). Dehulled rice seeds were rinsed in 70% (*w*/*v*) ethanol for 20 s and then in 2.5% (*w*/*v*) sodium hypochlorite for 20 min and washed three times in sterile distilled water. Surface-sterilized seeds were germinated on Murashige and Skoog media (Sigma-Aldrich, Saint Louis, USA) with 2% (*w*/*v*) sucrose and 0.8% (*w*/*v*) agar (pH 5.6) at 25 °C for 6 days. Healthy rice seedlings with uniform growth were sequentially cultivated in half and full-strength Yoshida solution [48] for one week and 12 days, respectively. For salt stress treatment, 25-day-old uniform rice seedlings were grown in full-strength Yoshida solution containing 0 (control) and 100 mM NaCl for 6 days. During the experiment, the solution was renewed every 3 days and adjusted to pH 5.5. Plants were grown in a growth chamber at 25 °C under 16 h light/8 h dark condition with 400 μmol m^−2^ s^−1^ light intensity.

After salt treatment for 6 days, we photographed rice seedlings and determined the growth parameters, photosynthetic parameters, and chlorophyll fluorescence parameters. Salt-treated rice seedlings were divided into shoots and roots. The fresh weights of shoots and roots were respectively determined and then samples were oven-dried at 80 °C to obtain the constant dry weight. Also, fresh samples were immediately snap-frozen in liquid nitrogen and stored at −80 °C for subsequent experiments. Dried samples were used for ion content determination. Each experiment was conducted with at least three biological replicates.

### 4.2. Pigment Content Determination

Chlorophyll and carotenoid were extracted from 0.1 g fresh leaves with 10 mL of 80% (*v*/*v*) acetone in the dark at room temperature until the leaves became blanched. Then, the pigments content was calculated by measuring the absorbance of the supernatant at 663, 646 and 470 nm with a UV-2550 spectrophotometer (Shimadzu, Kyoto, Japan) as previously described [49]. A blank sample containing only 80% (*v*/*v*) acetone was used for baseline correction.

### 4.3. Photosynthetic Parameters

The youngest fully expanded leaves were selected to measure photosynthetic parameters, including the *P*n, *T*r, *G*s, and *C*i, using an LI-6400 portable photosynthesis system (LI-COR, Lincoln, NE, USA) with a 2 × 3 cm leaf chamber system. Measurements were taken at a photosynthetic photon flux density (PPDF) of 400 μmol m^−2^ s^−1^, CO_2_ concentration of 400 μmol mol^−1^, with leaf temperature at 25.0 ± 0.5 °C and flow rate of 500 µmol s^−1^. Measurements were recorded after the stabilization of flux values after clamping onto new leaves. For rice seedlings, a single leaf cannot fill the entire leaf chamber system. Therefore, the relative area of the leaf was estimated by multiplying the measured leaf length by leaf width. Photosynthesis parameters were corrected based on the relative leaf area inside the chambers.

### 4.4. Chlorophyll Fluorescence Measurement

Chlorophyll fluorescence parameters were measured in the youngest fully expanded leaves of rice seedlings using a Dual-PAM 100 measuring system (Heinz Walz, Effeltrich, Germany). The fluorescence parameters included the F*v*/F*m*, Y(II), ETRII, 1-qP, NPQ and Y(NO). All plants were dark-adapted for 30 min before measurement.

### 4.5. Ion Content Measurement

Dried shoots and roots were ground into fine homogeneous powder using a ball mill (Retsch MM500, Haan, Germany) and weighed at 0.1 g each into a glass tube for measurement of Na^+^ and K^+^ contents. Then, a mixture of nitric acid/perchloric acid (4/1 *v*/*v*) was added to the glass tube and the solution was heated at 185 °C for 1.5 h using a digestion oven. The ion contents for Na^+^ and K^+^ of the samples were detected using an atomic absorption spectrophotometer (PerkinElmer PinAAcle 900 T, Waltham, MA, USA).

### 4.6. Lipid Analysis

Extraction buffer was added to 0.2 g of fresh leaves for lipid extraction as described previously [29]. After brief centrifugation, the chloroform phase was collected, then dried under N_2_ gas, and resuspended in 100 µL of 100% (*v*/*v*) high-performance liquid chromatography (HPLC)-grade chloroform purchased from Merck, Germany. Thin-layer chromatography was used to separate lipids in acetone/toluene/water (91/30/7, *v*/*v*/*v*) using silica gel 60 GF 254 plates purchased from Merck (Darmstadt, Germany), which were activated in 0.15 M (NH_4_)_2_SO_4_ and dried before usage [50]. Separated lipids were stained by spraying with 0.01% (*w*/*v*) primrose (Sigma, Saint Louis, USA) in 80% (*v*/*v*) acetone and visualized under UV light. Then, the scraped silica gel of each lipid was transmethylated at 80 °C for 25 min with methanol HCl solution containing pentadecanoic acid (C15:0, Sigma, Saint Louis, USA) as an internal standard. The phase was separated with 0.9% NaCl_2_ (*w*/*v*) and hexane, and the organic phase was dried with N_2_ gas and resuspended in 50 µL hexane. Samples (2 μL) of the hexane phase were analyzed by gas chromatography (GC-2010; Shimadzu, Kyoto, Japan) equipped with a flame ionization detector. The gas chromatograph was set to an initial temperature of 140 °C for 2 min, followed by a 25 °C min^−1^ ramp to 160 °C, then an increase of 8 °C min^−1^ to 240 °C, and maintained at this temperature (total run time, 58 min). The double bond index was calculated as DBI = [(16:1 mol%) × 1 + (16:2 mol%) × 2 + (16:3 mol%) × 3 + (18:1 mol%) × 1 + (18:2 mol%) × 2 + (18:3 mol%) × 3]/100 [51].

### 4.7. Determination of Lipid Peroxidation and Membrane Integrity

Lipid peroxidation was assessed based on levels of MDA. The MDA content was determined by the thiobarbituric acid (TBA) method and by spectrophotometry at 450, 532, and 600 nm according to the previous method [52]. A blank sample containing only buffer was used for the baseline correction. Briefly, pre-cooled 10% (*v*/*v*) trichloroacetic acid (TCA) was added to 0.1 g fresh leaves ground to powder in liquid nitrogen. The supernatant was mixed with an equal amount of 0.67% (*w*/*v*) TBA and boiled for 30 min.

The membrane integrity was examined by measuring EL as follows [53]: 0.1 g fresh leaf discs were incubated in 10 mL deionized water, and conductivity was measured before (EC1) and after boiling (EC2) using a Horiba B-173 conductivity meter (Horiba, Japan). The percentage of EL was calculated as EC1/EC1.

### 4.8. Statistical Analysis

Statistical analysis was performed using one-way ANOVA in SPSS software version 20.0 (SPSS Inc., Chicago, IL, USA). LSD testing was used to compare significant differences between samples (*p* < 0.05). All the data were expressed as the mean ± standard deviation (SD).

## 5. Conclusions

The overexpression of *OsMGD1* facilitates membrane lipid remodeling, bolstering membrane integrity and fluidity. This, in turn, improves the capture, transfer, and fixation of light energy, ultimately enhancing plants’ salt tolerance. Conversely, the suppression of *OMGD1* expression leads to reduced synthesis of MGDG and DGDG, causing considerable damage to photosynthetic membranes and intensifying the deleterious effects of salt stress. Our findings demonstrate that beyond regulating Na^+^/K^+^ homeostasis, *MGD* improves salt tolerance in rice by optimizing photosynthetic efficiency and reinforcing membrane stability. In summary, this study elucidated the physiological and molecular functions of *OsMGD1* in improving salt tolerance in rice, which provides an important theoretical basis for further exploring the mechanism of crops responding to salt stress.

## Data Availability

Data are contained within the article and Supplementary Materials.

## Supplementary Materials

The following supporting information can be downloaded at: https://www.mdpi.com/article/10.3390/plants13111474/s1, Figure S1: Effects of *OsMGD1* on shoot fresh weight (**a**), root fresh weight (**b**), total fresh weight (**c**) and total dry weight (**d**) of rice under control or salt stress (100 mM NaCl for 6 d) condition; Figure S2: Analysis of (**a**) chlorophyll *a* (Chl *a*), (**b**) chlorophyll *b* (Chl *b*), and (**c**) intercellular CO_2_ concentration (*C*i) parameters of WT and *OsMGD1* transgenic rice under salt stress at the seeding stage; Figure S3: Changes of DBI in MGDG (**a**) and DGDG (**b**) under salt stress.
