# Supplementary material for: OsMGD1-Mediated Membrane Lipid Remodeling Improves Salt Tolerance in Rice
Source: Plants (Basel). 2024 May 27;13(11):1474. doi: 10.3390/plants13111474 (PMC11174947; doi:10.3390/plants13111474)
Supplement: Supplementary file 1 [file plants-13-01474-s001.zip › plants-2978603-supplementary.pdf]

Supporting Information

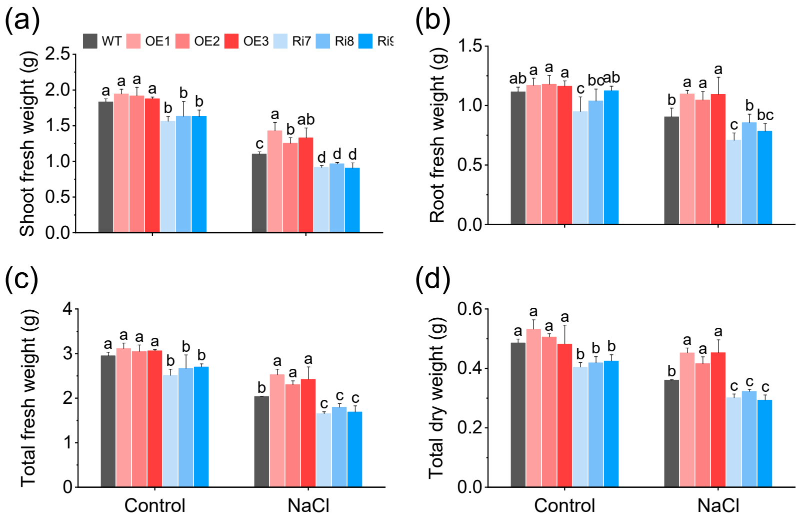

Figure S1. Effects of *OsMGD1* on shoot fresh weight (**a**), root fresh weight (**b**), total fresh weight (**c**) and total dry weight (**d**) of rice under control or salt stress (100 mM NaCl for 6 d) condition. WT, wild type; OE, *OsMGD1*-overexpressing lines; Ri, *OsMGD1*-RNA interfering lines. Data are means ± SD (n = 5 biological replicates). Different letters indicate significance at $p < 0.05$ as determined by LSD-test.

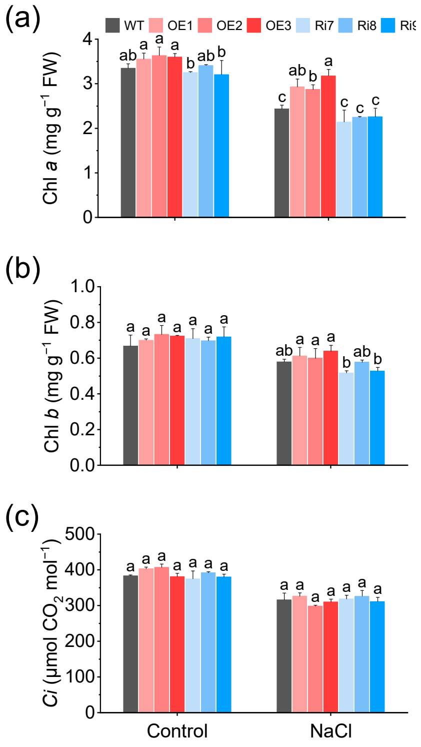

Figure S2. Analysis of (**a**) chlorophyll a (Chl *a*), (**b**) chlorophyll b (Chl *b*), and (**c**) intercellular CO_2 concentration (Ci) parameters of WT and *OsMGD1* transgenic rice under salt stress at the seeding stage. WT, wild type; OE, OsMGD*1*-overexpressing lines; Ri, *OsMGD1*-RNA interfering lines. Data are means ± SD (n = 5 biological replicates). Different letters indicate significance at $p < 0.05$ as determined by LSD-test.

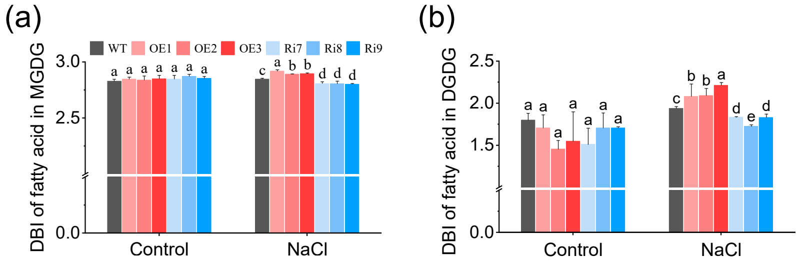

Figure S3. Changes of DBI in MGDG (**a**) and DGDG (**b**) under salt stress. DBI, double-bond index; MGDG, monogalactosyldiacylglycerol; DGDG, digalactosyldiacylglycerol. WT, wild type; OE, *OsMGD1*-overexpressing lines; Ri, *OsMGD1*-RNA interfering lines. Data are means ± SD ($n = 3$ biological replicates). Different letters indicate significance at $p < 0.05$ as determined by LSD-test.
